# Real-time observation of functional specialization among phosphorylation sites in CFTR

**DOI:** 10.1085/jgp.202213216

**Published:** 2023-01-25

**Authors:** Daniel T. Infield, Miranda E. Schene, Frederico S. Fazan, Grace D. Galles, Jason D. Galpin, Christopher A. Ahern

**Affiliations:** 1 Department of Molecular Physiology and Biophysics and Iowa Neuroscience Institute, University of Iowa, Carver College of Medicine, Iowa, IA, USA

## Abstract

Phosphoregulation is ubiquitous in biology. Defining the functional roles of individual phosphorylation sites within a multivalent system remains particularly challenging. We have therefore applied a chemical biology approach to light-control the state of single candidate phosphoserines in the canonical anion channel CFTR while simultaneously measuring channel activity. The data show striking non-equivalency among protein kinase A consensus sites, which vary from <10% to >1,000% changes in channel activity upon phosphorylation. Of note, slow phosphorylation of S813 suggests that this site is rate-limiting to the full activation of CFTR. Further, this approach reveals an unexpected coupling between the phosphorylation of S813 and a nearby site, S795. Overall, these data establish an experimental route to understanding roles of specific phosphoserines within complex phosphoregulatory domains. This strategy may be employed in the study of phosphoregulation of other eukaryotic proteins.

## Teaser

Infield and co-authors introduce an approach whereby the individual functional contribution of phosphoserine residues can be observed in real time via site-specific encoding of a caged-serine unnatural amino acid.

## Introduction

Phosphorylation is an essential and ubiquitous mode of functional regulation and subcellular localization for many ion channels including voltage-gated cation channels (Roybal et al., 2020), ligand-gated channels (Nakamura et al., 2015), and CFTR, the ion channel whose dysfunction causes cystic fibrosis (Rowe et al., 2005). The functional consequence of phosphorylation ranges from subtle changes in gating equilibria to (in the case of CFTR) a strict dependence on phosphorylation for activity (Cheng et al., 1991).

**Figure 1.**
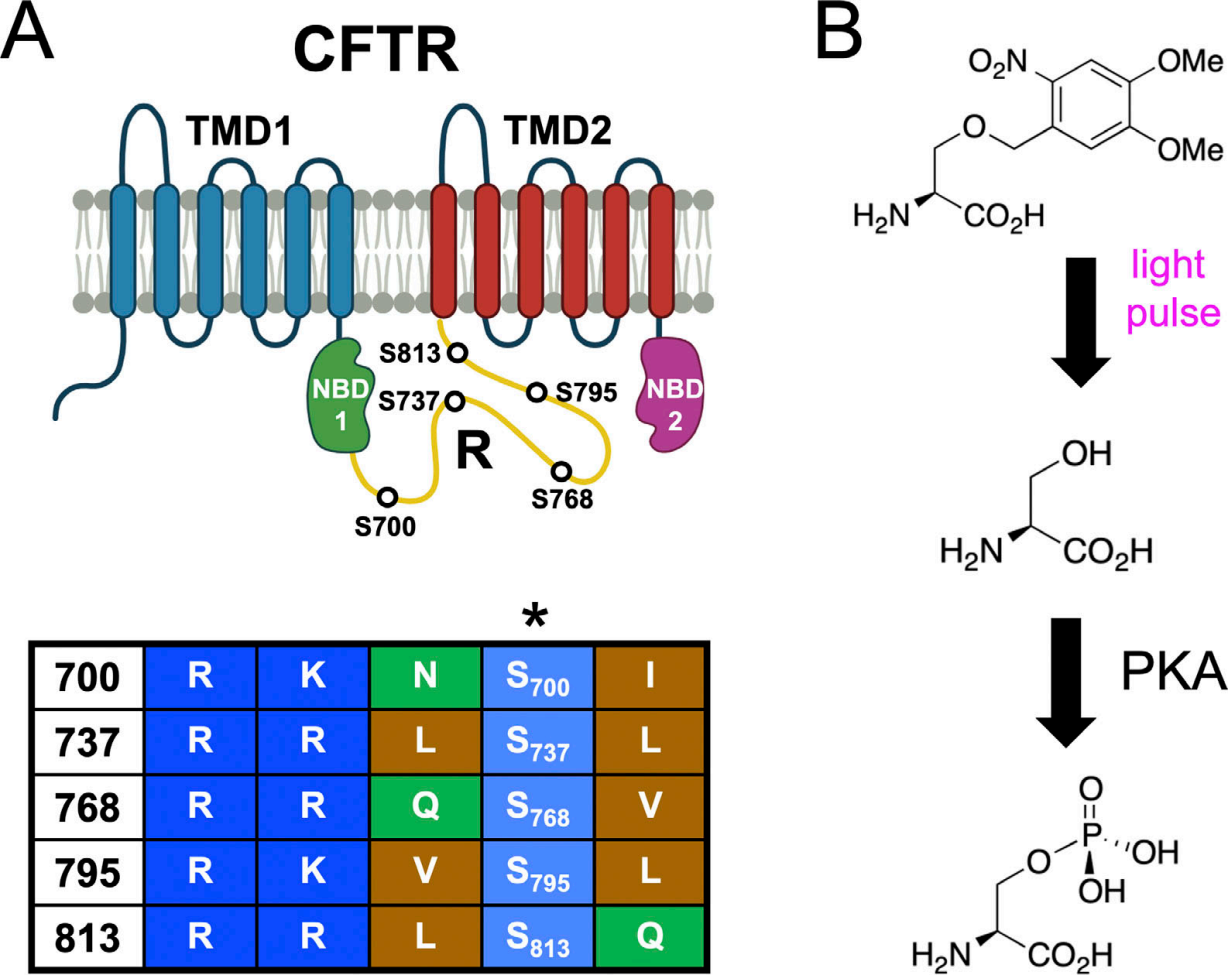
**A chemical biology approach to study CFTR phosphorylation. (A)** Top: Cartoon domain representation of CFTR showing the R domain in gold. Bottom: The serine residues interrogated in this study are numbered. **(B)** Chemical representation of photo-conversion of the encoded caged serine (Ser^cage^) residue to native serine, which is then available for in vivo phosphorylation by PKA.

In common with other channels, there exists biochemical evidence in vivo and in vitro that CFTR is phosphorylated by PKA at multiple sites (Rich et al., 1993). Biochemical (Mense et al., 2006) and structural (Liu et al., 2017; Zhang et al., 2018) approaches have led to a general hypothesis that phosphorylation of the R domain leads to an overall disengagement of this domain from the rest of the channel, thereby allowing ATP-dependent dimerization of nucleotide binding domains (NBDs) and channel gating to proceed. However, functional evidence also suggests that the phosphorylated R domain stimulates CFTR activity beyond that allowed by its deletion (Winter and Welsh, 1997). Functional data from conventional site-directed mutagenesis suggests a degree of non-equivalency among sites, with most sites inferred to be stimulatory but at least two (S737 and S768) that may be inhibitory (Vais et al., 2004; Wilkinson et al., 1997, but see Hegedus et al., 2009). While informative, there are obvious limitations with the use of natural mutagenesis since alanine and glutamate are, at best, structural approximations of non-phosphorylated and phosphorylated serine. This challenge appears to be particularly relevant to CFTR as some biochemical data have suggested that alanine mutations may actually structurally mimic the phosphorylated R domain more closely than the non-phosphorylated R domain (Dulhanty et al., 1995). Similar challenges have been observed when studying phosphoregulation of potassium channels, wherein the mutation of candidate phosphoserines to alanine caused a functional effect similar to that of phosphorylation by PKA (Drain et al., 1994). Furthermore, since point mutation is permanent, mutagenesis will not yield kinetic information about phosphorylation. For CFTR, the kinetics of phosphorylation of individual PKA consensus sites have been measured via NMR of the isolated R domain in solution (Bozoky et al., 2017). These data suggest a rank order of phosphorylation rates that is generally supported by data from the intact channel, attained with antibodies raised against a few select PKA CFTR consensus sites (Hegedus et al., 2009). To directly monitor the functional consequence of phosphorylation of individual serine residues within the multivalent system of the CFTR R domain, we applied a chemical biology approach that enabled the encoding of a O-(4,5-dimethoxy-2-nitrobenzyl)-L-serine (DMNB), a.k.a. caged-serine (Ser^cage^), within the channel (Fig. 1 A) expressed in the human HEK cell line. This so-called “unnatural” amino acid is rapidly converted to native serine via a controlled pulse of short wavelength light (Fig. 1 B; Lemke et al., 2007).

Using this method, we observed striking functional diversity among PKA consensus sites in both the rate and degree of functional effect of their phosphorylation, and we uncovered evidence that the phosphorylation of key stimulatory sites S795 and S813 is strongly coupled.

## Materials and methods

### Cell culture

HEK 293T cells were cultured in DMEM high glucose + 10% FBS, 1% pen-strep, and 1% L-glutamine. We utilized the variant CRL 3216, which we received directly from ATCC.

### DNA constructs

We received pANAP from Peter Schultz via Addgene and generated pCAGEDSER (the plasmid encoding the tRNA and synthetase for caged serine) by introducing the relevant active site mutations for the recognition of caged serine (Lemke et al., 2007) via gblock (IDT). pCAGEDSER was propagated in Stbl3 cells at 30°C. pCMV-CFTR was a gift from Paul McCray (University of Iowa, Iowa City, IA). Note that this version has methionine at the common polymorphism positions 470 and 1475. The existing TAG stop codon was exchanged to TAA, and then TAG stop codons at R domain sites of interest were introduced via gblock (IDT). The pCDNA_eGFP_152TAG plasmid carries an HA tag.

### Electrophysiology and photochemistry

To encode caged serine into CFTR, 10-cm dishes of HEK cells were co-transfected with ∼1.1–1.5 μg of CFTR, 3.5 μg pCAGEDSER plasmid, and 0.5 μg eGFP_152TAG plasmids using PolyJET in the presence of 1.5 mM caged serine amino acid. Cells were seeded to 35-mm dishes for patch clamp using Versene, a non-enzymatic dissociation solution of 0.5 mM EDTA in divalent cation-free PBS. Recordings (green, healthy cells) took place 1–2 d after transfection. Bath solution contained 90 mM NaCl, 2 mM CsCl, 1 mM MgCl_2_, 1 mM CaCl_2_, 10 mM HEPES, and 110 mM Mannitol, and pH was adjusted to 7.4 with NaOH. Pipette solution contained 120 mM L-Asp, 20 mM CsCl, 1 MgCl_2_, 5 mM EGTA, 10 HEPES, 120 mM CsOH, and pH was adjusted to 7.2 with CsOH. Before use, 5 mM ATP was added to pipette solution, pH was corrected with CsOH, and the solution was frozen in aliquots at −30°C. forskolin was obtained from Sigma-Aldrich, and CFTR inh 172 was obtained from Sigma-Aldrich or Cayman Chemicals.

Light was delivered via Thorlabs LED 385 nm, model number M385LP1-C5, driven by the LEDD1B power supply (Thorlabs) through the 20× objective of the microscope and shuttered via TTL pulse sent to LEDD1B by the digital output of Clampex. In comparing the fold change of current among variants, the method carries the intrinsic assumption that the same caged serine residue encoded in different positions is decaged with similar efficiency. All cells included for analysis had max current signal at least 75% blocked by CFTR inh172. CFTR currents were ramped once every second. Note that for S768TAG recordings, the effects of decaging were very small and thus potentially affected by any deviations from a perfectly flat plateau immediately before the light pulse. For this mutant, the decaging traces were baseline-corrected to 30 s preceding the pulse. Fitting was performed in Clampfit using the exponential (standard) function (first or second order) with the exception of one trace from S795TAG, for which we utilized the exponential (alpha) function (second order) as a means to seed floating values and achieve a good fit.

### Western blotting

For each condition, a 10-cm dish of HEK cells was transfected with 1.5 μg CFTR, 3.5 μg pCAGEDSER, and 0.5 μg GFP 152TAG in the presence or absence of caged serine (Cat# 61935; Asta-Tech). Cells were harvested on ice ∼2 d after transfection and flash frozen in liquid nitrogen. Pellets were lysed in RIPA buffer (Sigma-Aldrich) plus protease inhibitors (Roche). Nuclei and cell debris were cleared via centrifugation at 4°C. Protein was measured via Bradford assay and 60 µg of lysate or a dilution thereof was run on 4–20% TGX gel (Biorad) and transferred to nitrocellulose. CFTR was detected by AB 596 (Cystic Fibrosis Foundation; 1:1,750). Membranes were stripped using Restore stripping buffer (Cat# 21059; Thermo Fisher Scientific). β-Actin was detected by use of HRP-conjugated AC-15 antibody (Novus Biologicals). Blots were visualized with ECL (Clarity, Biorad).

### Whole-dish photochemistry

**Figure S1.**
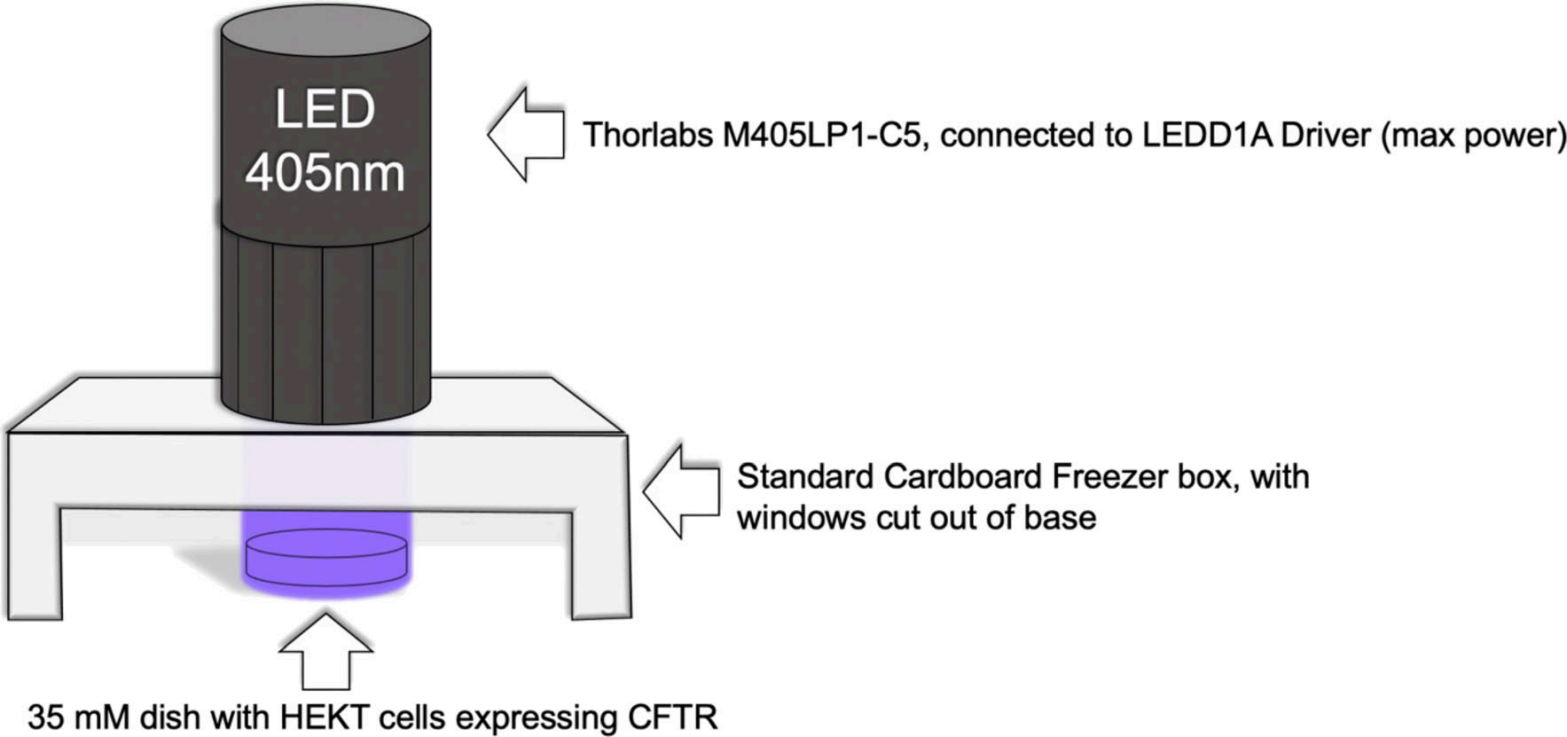
Diagram showing the composition of the whole-dish photochemistry apparatus.

HEK 293T cells in 35-mM dishes were transfected with 0.25 μg WT-CFTR-TAA plus 0.75 μg WT HA eGFP, or with 0.22 μg S795TAG-CFTR plus 0.7 μg pCAGEDSER and 0.1 μg eGFP-152TAG. To maximize consistency, the dishes were transfected as appropriate master mixes. Approximately 40 h after transfection, media was exchanged with DPBS plus calcium and magnesium (Gibco) supplemented with 8 g/liter glucose, with or without 10 μM forskolin to stimulate PKA. After 5 min, dishes were either exposed to a blue light LED (405 nm, Thorlabs; Fig. S1) for 3 min or without light in parallel. After this treatment, dishes were incubated at room temperature for 2 additional min and directly lysed on ice in ice-cold 2X LSB (Biorad) supplemented with 1 μl/ml Pierce Universal nuclease. Samples were flash frozen and stored at −80°C. For each condition, an equal volume of sample (lysate) was loaded and subjected to Western blotting using the conditions described above, except that ab217 (Cystic Fibrosis Foundation) was used, which recognizes the unphosphorylated S813 PKA motif within CFTR. Densitometry was performed in ImageJ.

### Expression and purification of PKA-catalytic subunit

pET15b PKA Cat (PKA encoding plasmid) was a gift from Susan Taylor (plasmid# 14921; Addgene; http://n2t.net/addgene:14921; RRID:Addgene_14921). PKA plasmid was expressed in BL21 cells, which were grown in 1.2 liters LB Media supplemented with carbapenem at 37°C. When culture density reached 0.4–0.6 OD, IPTG was added to a final concentration of 0.1 mM, and the temperature was reduced to 30°C for 8 h. 10 g of bacterial cells were harvested and resuspended in 100 ml lysis buffer (50 mM NaH_2_PO_4_, 250 mM NaCl, 1 mM DTT, Roche complete mini EDTA-free protease inhibitors, and 0.25 mM PMSF, pH 7.4). The resuspended cells were lysed by sonication. The soluble fraction was separated from the insoluble fraction via centrifugation at 15,000 × *g*. 105 ml of clarified lysate was collected, and 6 ml of washed and packed TALON resin was added. The resin was allowed to equilibrate for 30 min at 4°C. After equilibration, the resin was separated into 3 × 2 ml fractions. Each fraction was washed once with 10 ml lysis buffer, twice with 15 ml wash buffer A (50 mM NaH_2_PO_4_, 250 mM NaCl, and 1 mM DTT, pH 7.4), three times with 15 ml wash buffer B (50 mM Tris, 50 mM NaCl, and 1 mM DTT, pH 7.4), and three times with 15 ml wash buffer C (50 mM Tris, 50 mM NaCl, 25 mM Imidazole, and 1 mM DTT, pH 7.4). Each fraction was eluted with 5 ml elution buffer (50 mM Tris, 50 mM NaCl, and 300 mM Imidazole, pH 7.4), and the 5 ml fractions were concentrated to 3 ml in 10 kD cutoff Amicon centrifugal filters. The fractions were subjected to size exclusion chromatography using S200 Sephadex in 50 mM TRIS, 100 mM NaCl, 10% Glycerol, and 1 mM DTT, pH 7.4. Peak fractions of PKA-C from SEC were concentrated and flash frozen.

### Peptide synthesis

The peptides PKS and PKS^(caged)^ were synthesized by standard Fmoc solid-phase peptide methods on a Liberty Blue peptide synthesizer (CEM Corporation) on a 0.025 mmol scale with C-terminal amides. Fmoc-DMNB-serine was synthesized from DMNB-serine (AstaTech) and Fmoc-Cl by standard procedure. Deprotection of peptides and removal from resin with trifluoroacetic acid and precipitation with diethyl ether resulted in chalky solids, which were then purified via preparative HPLC (Waters Corporation). Fractions containing the peptides of interest were combined and lyophilized to white (PKS) and pale yellow (PKS^(caged)^) fluffy residues, ∼15 mg each. High-resolution mass spectrometry was performed on a Waters QToF Premier Quadrupole instrument, in positive mode, and the expected masses were observed as multiply-charged species.

**Figure S2.**
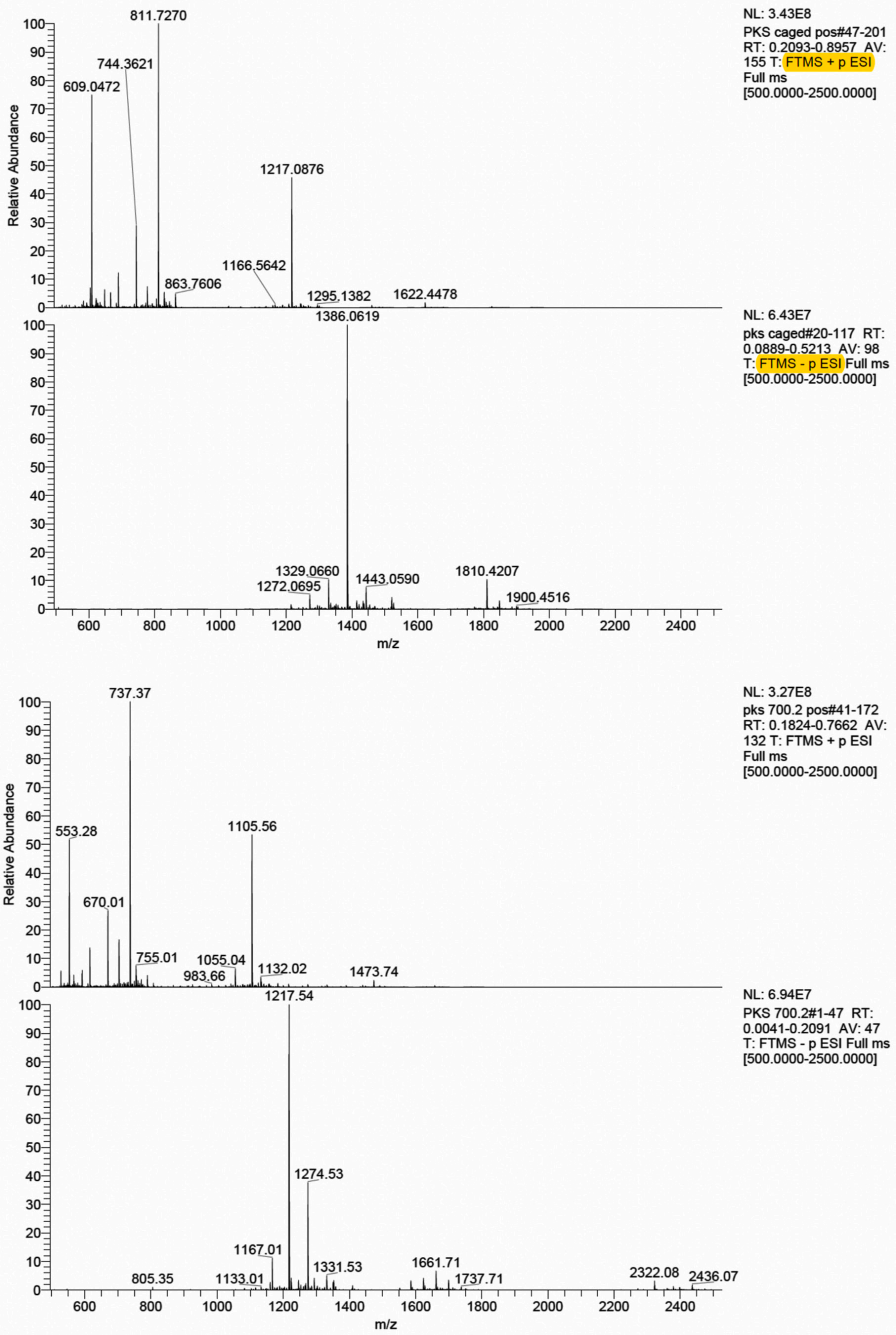
Full ESI mass spectra for the PKS^(caged)^ and PKS peptides.

The sequence of PKS TTYADFIASGRTGRRNSIHD expected mass 2,209.4; found mass 2,209.1. Sequence of PKS^(caged)^ TTYADFIASGRTGRRNS(cage)IHD expected mass 2,432.3; found mass 2432.2. (Fig. S2).

### Surface plasmon resonance

The Biacore T200 instrument was used to conduct all experiments with a CM5 chip at 25°C. PKA-C (13 μM stock concentration) was used as a ligand to capture/immobilize on the CM5 chip surface. PKI, PKS, and PKS^(caged)^ at 10 mM stock concentration were used as analytes to flow over the ligand-immobilized surfaces. Flow Cell (FC) 1 was used as the reference for FC2.

PKA-C was diluted (1:100 dilution, 0.13 µM diluted concentration) in 10 mM sodium acetate buffer at pH 5.5 and immobilized onto FC2 to a level of ∼7,300 RU using standard amine coupling chemistry. HBS-P (10 mM HEPES, pH 7.4, 150 mM NaCl, and 0.05% v/v surfactant P20) was used as the immobilization running buffer.

Kinetics experiments were performed for all analytes in the presence of HBS-P supplemented with 0.2 mM AMP-PNP, and 2 mM MgCl_2_. One 20-s pulse of 1 M NaCl solution was injected for surface regeneration. The flow rate of all analyte solutions was maintained at 50 μl/min. The contact and dissociation times used for all analytes were 120 and 360 s, respectively. For each run, analyte solutions were injected in duplicate. Sensorgrams from the overnight kinetics were evaluated by using 1:1 kinetics model fitting in BiaEvaluation Software. Note that a small component of nonspecific binding at high concentrations of peptides is suggested by the failure of the signals to fully saturate at 10 μM (see Fig. 3), and thus the derived affinity values may be slightly underestimated.

### Online supplemental material

Fig. S1 contains an illustration of the experimental setup used for whole-dish photochemistry. Fig. S2 shows confirmatory mass spectrometry results for the two PKA substrate peptides synthesized in-house (PKS and PKS^(^^caged)^).

## Results

### Slow phosphorylation of S813 governs the activity of CFTR

**Figure 2.**
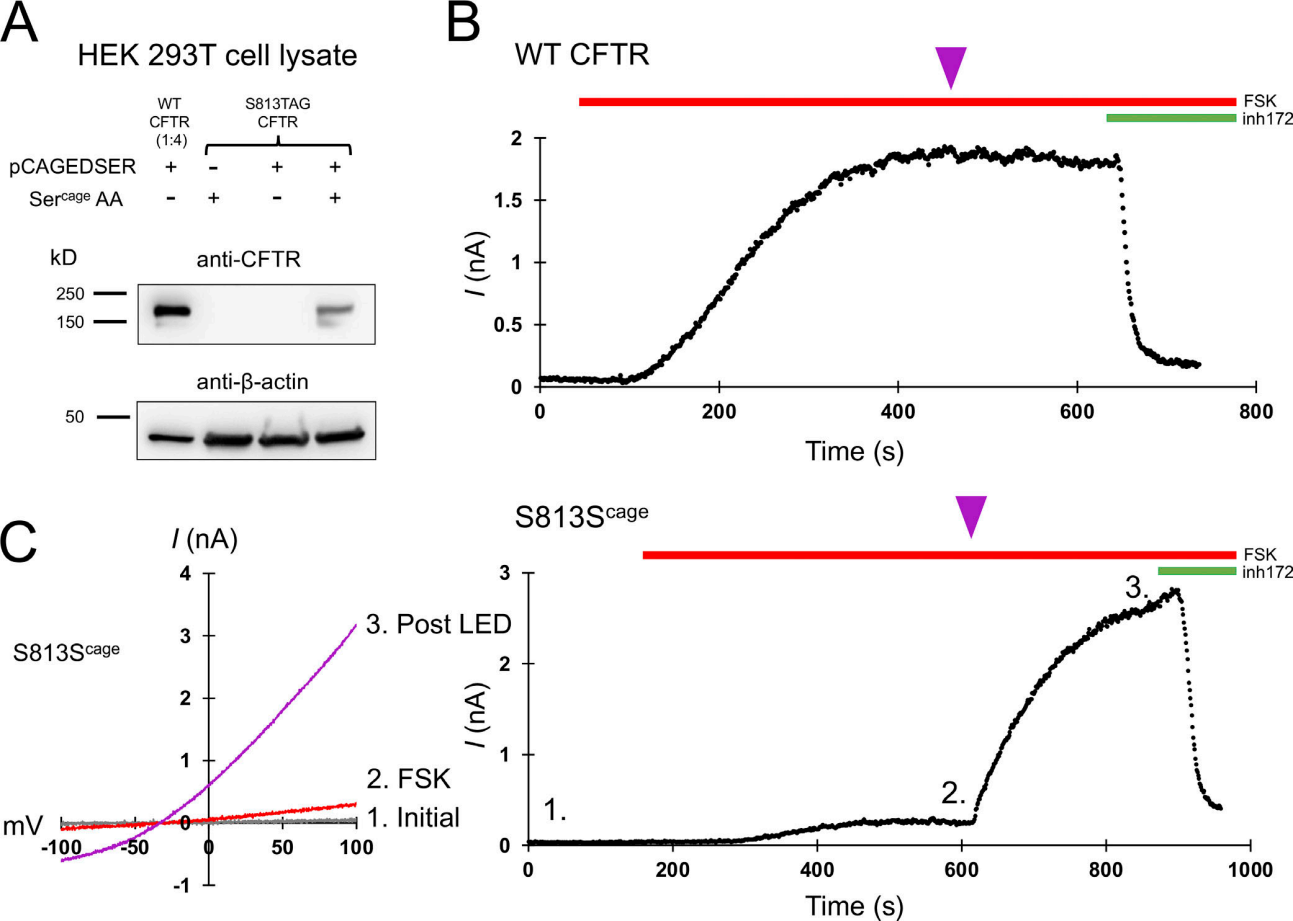
**CFTR gating requires S813 phosphorylation. (A)** Western blot using the CFTR C-terminal antibody ab596 showing specific encoding of caged serine at position S813TAG. The WT sample was diluted 1:4 for comparison to the S813 condition. pCAGEDSER refers to the plasmid encoding the tRNA and synthetase, whereas Ser^cage^ AA indicates the addition of the caged serine amino acid. **(B)** Whole-cell WT CFTR current elicited by 10 μM forskolin (FSK) and plotted at +80 mV. The purple arrowhead indicates 250 ms of LED exposure. **(C)** IV curves (left) and plotted current of whole-cell S813S^cage^ CFTR current over time (right). Conditions correspond to 1: initial, 2: after forskolin addition, and 3: post-light pulse exposure. *n* = 3 experimental replicates for both WT and S813S^cage^ variants; quantification in Fig. 6 and Table 1. Unlike WT CFTR, S813S^cage^ CFTR current was highly potentiated by the pulse of light. Source data are available for this figure: SourceData F2.

To site-specifically encode caged serine into CFTR in HEK 293T cells, we made use of a previously identified orthogonal caged serine synthetase and tRNA pair, which here we refer to as pCAGEDSER (Lemke et al., 2007). Using Western blotting with a C-terminal CFTR antibody, we observed specific encoding of caged serine into position S813 of CFTR expressed in HEK cells (Fig. 2 A). The use of cultured mammalian cells enabled the delivery of light through the objective of the inverted microscope used for the patch clamp.

While under whole-cell voltage clamp, we activated these channels with 10 μM forskolin, a dose which generates maximum cAMP levels in HEK cells (Gilissen et al., 2015), and supports robust activation (and thus PKA mediated phosphorylation) of WT CFTR (Hegedus et al., 2009). A single 250-ms pulse of 385 nm light was delivered through the microscope objective from an LED (see Materials and methods) at the current plateau, i.e., after other sites have reached apparent phosphoryl-equilibrium from in vivo PKA signaling (Fig. 2, magenta arrowheads). This dose of light caused a negligible effect on WT CFTR (Fig. 2 B, summary data in Fig. 6, and Table 1). Forskolin stimulation of cells expressing S813S^cage^-CFTR led to a very small initial CFTR current, while the delivery of a light pulse after the current plateau (to decage S813) led to a massive increase in CFTR current over minutes (τ of 161 ± 68.8 s; Fig. 2 C). This increase was variable from cell to cell but averaged 12.1 ± 0.9-fold (*n* = 3; summary data in Fig. 6 and Table 1). Since decaging reveals the native serine residue at S813, thus converting the channel to wild type, this magnitude of effect from decaging suggests that when this site is unavailable for phosphorylation, the channel is very weakly (<10%) active relative to WT-CFTR. The time course of the increase in channel activity observed upon decaging reasonably reflects the rate of phosphorylation for S813 in vivo as determined by a site- and phospho-specific antibody (Hegedus et al., 2009).

**Table 1.** Functional changes in whole cell CFTR in response to decaging pulse

CFTR variant	Fold change (relative to 1)		Kinetics (τ, seconds)		*n*
	Value	SD	Value	SD	
WT	**0.995**	0.010	N/A	N/A	3
S700(cagedser)	**1.111**	0.099	**17.3**	2.4	3
S737(cagedser)	**2.542**	0.978	**38.7**	19.2	3
S768(cagedser)	**0.934**	0.034	**12.9**	2.0	3
S795(cagedser)	8.385	4.664	t_1_ = **11.4** t_2_ = 155.6	t_1_ = 1.1 t_2_ = 108.2	4
S813(cagedser)	12.055	0.893	161.0	68.8	3

Bolded values are P < 0.05 by Student’s *t* test (unpaired) compared to that of S813(cagedser).

### Caging serine drastically reduces the affinity of PKA for its recognition motif

**Figure 3.**
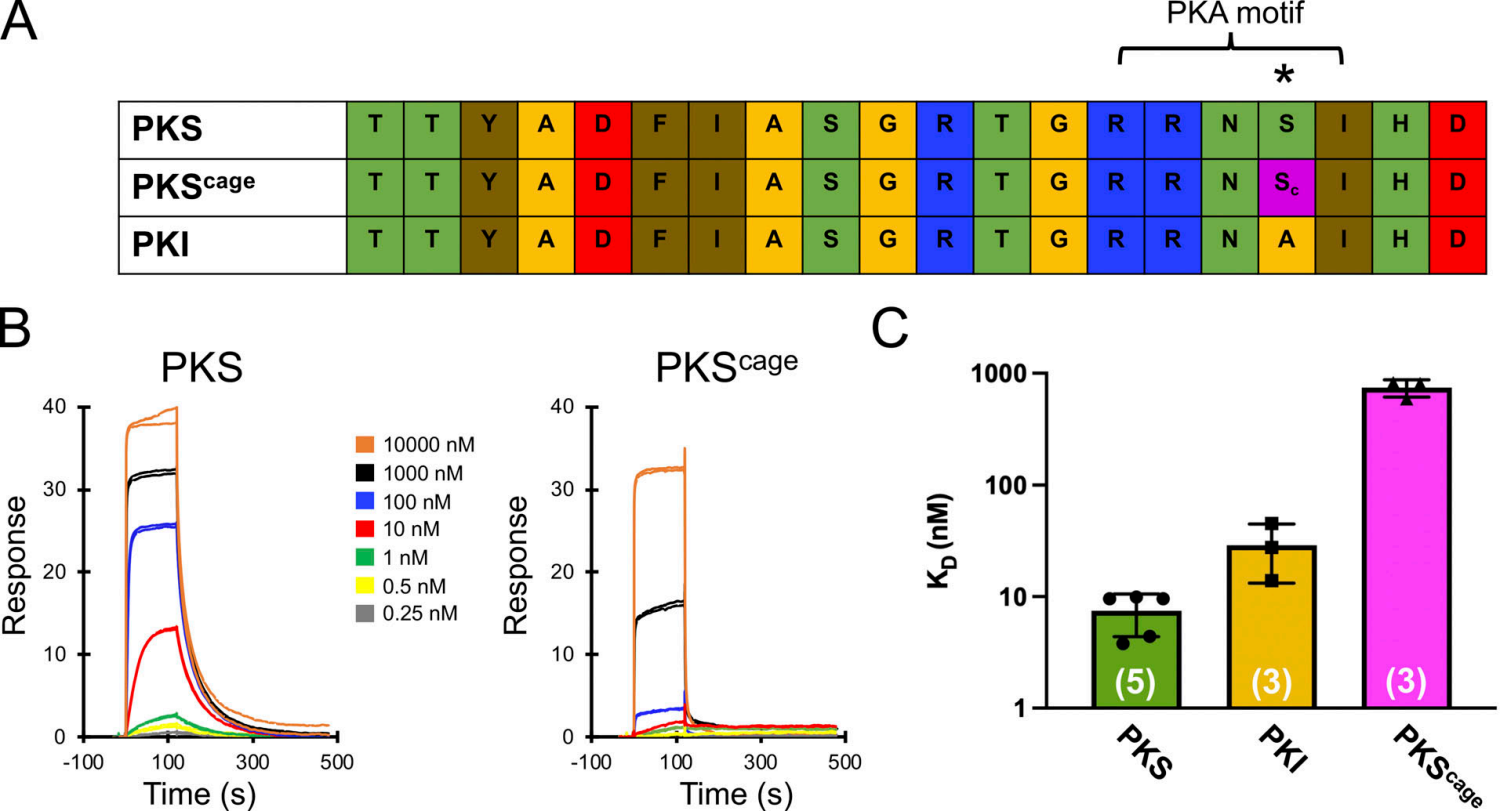
**Effect of caging serine on interaction of substrates with the catalytic subunit of PKA. (A)** Sequence of peptides used for binding studies. The asterisk is over the position of the phosphoserine or relevant mutation. **(B)** Example surface plasmon resonance sensorgrams for the indicated peptide (as analyte) in the presence of the poorly hydrolysable ATP analog AMP-PNP. **(C)** Graph of K_D_ values derived from SPR assays; please note the log_10_ scale at the left axis. P values for unpaired *t* test against PKS^(caged)^ are 0.009 and 0.010 for PKS and PKI, respectively, with the number of experimental replicates in parentheses.

Recent functional work suggests that activation of CFTR can result from simple binding of PKA to CFTR without residue phosphorylation, and that this effect is preserved (at least partially) in alanine and glutamate mutant channels (Mihalyi et al., 2020). Data from our photo-decaging data above establish that a change in the interaction of PKA with a specific CFTR phosphorylation site is associated with a large increase in channel activity. The cage ensures that the serine of interest cannot be phosphorylated, but the increase in current upon decaging may be associated with PKA binding, phosphorylation, or both. To measure whether the cage affects the affinity of a consensus site for PKA, we measured explicit binding constants via surface plasmon resonance (SPR). We used the poorly hydrolyzable ATP analog AMP-PNP and Mg^2+^ to structurally mimic the formation of the associated Michaelis–Menten complex (Knape et al., 2015). For substrates, we used variants of PKI_5-24_, a peptide comprising the region of the heat-stable PKA inhibitor (PKI) that mediates high-affinity interaction (Glass et al., 1989). This peptide has been demonstrated to be a useful model for the characterization of PKA–substrate interactions via SPR (Knape et al., 2015; Glass et al., 1989). Experiments were done on PKI_5-24_, PKS (a variant of PKI_5-24_ wherein the naturally occurring alanine at the phosphoserine site is mutated to serine), and PKS^(caged)^, wherein caged serine was encoded at the same position (Fig. 3 A). In the presence of AMP-PNP and Mg^2+^, PKS bound with high affinity (K_D_ of ∼7.4 nM, *n* = 5; Fig. 3, B and C). Photo-caging the serine caused a profound effect on affinity for PKA, reducing it by ∼100-fold (∼740 nM, *n* = 3). By comparison, PKI (with an alanine mutation at the position of the serine) demonstrated a K_D_ of ∼30 nM (*n* = 3), approximately fourfold lower than PKS, which suggests that side chain elimination only mildly impedes PKA binding. While it is difficult to relate these in vitro data directly to the in vivo PKA–CFTR interaction*,* the relative differences lead us to conclude that caging of the serine strongly impedes PKA binding to consensus sites, as well as preventing phosphorylation.

### Decaging of PKA consensus sites in the middle of the R domain is associated with diverse functional effects

**Figure 4.**
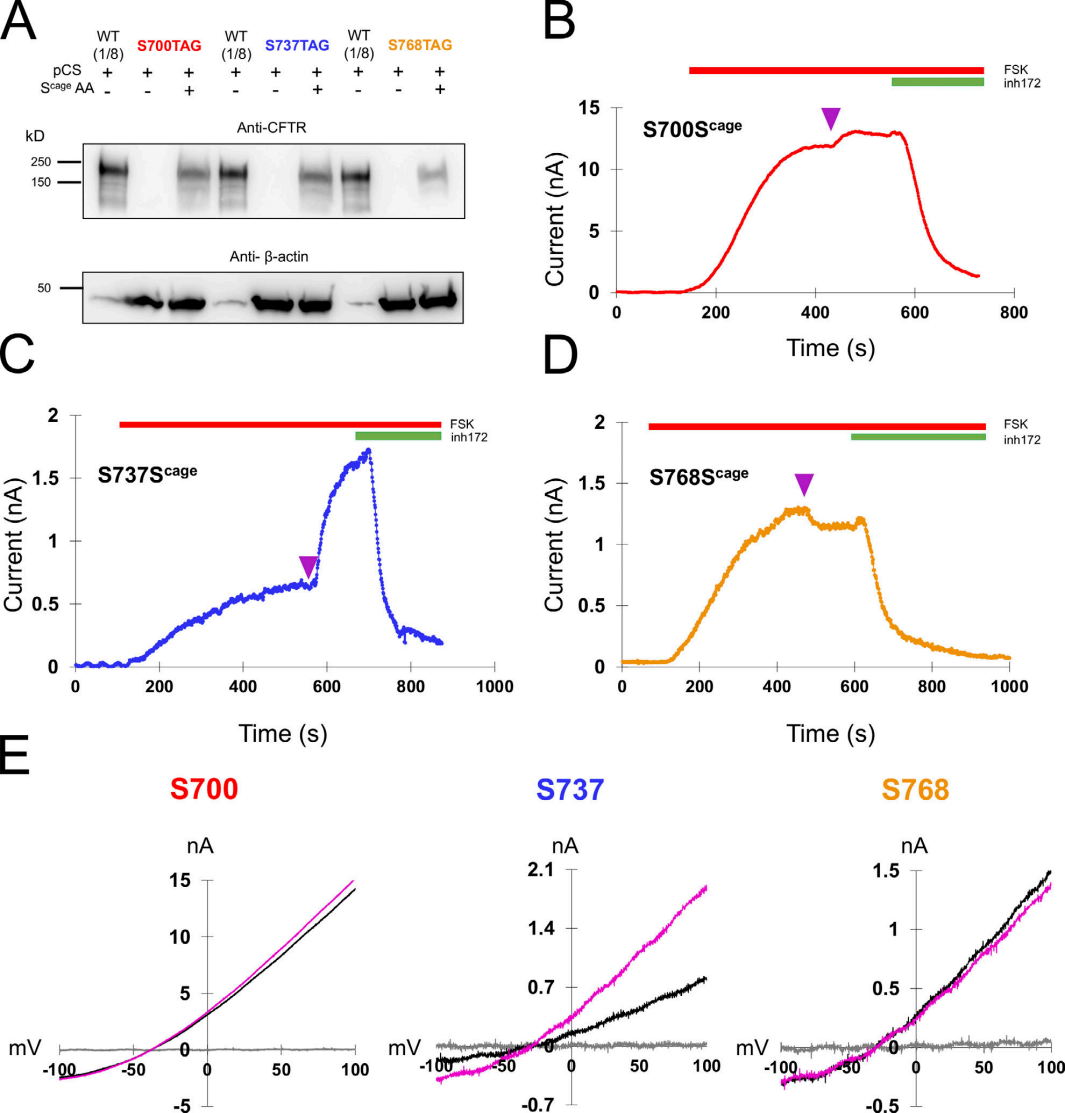
**Decaging of middle sites in the R domain yield fast, diverse functional effects. (A)** Western blot showing the specific rescue of S700TAG, S737TAG, and S768TAG-CFTR with caged serine via pCAGEDSER (pCS) synthetase. The WT CFTR lysate was diluted 1:8 to compare with rescued samples. **(B–D)** Representative traces of HEK cells expressing CFTR channels with caged serine encoded at S700, S737, or S768, with decaging occurring at plateau (purple arrowhead). *n* = 3 experimental replicates for each variant; quantification in Fig. 6 and Table 1. **(E)** IV curves for the individual cells in C–E: gray = initial, black = plateau of forskolin, magenta = after light pulse. Source data are available for this figure: SourceData F4.

In addition to S813, we separately encoded caged serine at S700, S737, and S768 (Fig. 4). At these sites, we observed diverse functional effects. At S700 (Fig. 4 B), a small degree of stimulation (1.11 ± 0.099-fold, *n* = 3) was observed after decaging. The rate was remarkably fast, with a τ of 17.3 ± 2.4 s. The magnitude of the effect was greater for S737 wherein we observed a 2.5 ± 0.97-fold increase on average (*n* = 3), with a τ of 38.7 ± 19.2 s (Fig. 4 C). Interestingly, S737 was recently identified as a site of human mutation (S737F) associated with a mild form of cystic fibrosis (Terlizzi et al., 2018). This is consistent with our observation that CFTR is substantially active even when this site is not phosphorylated. Finally, decaging of S768 was associated with a small decrease in current (fold change of 0.93 ± 0.033,*n* = 3). The rate was fast, with a τ of 12.9 ± 2.0 s.

Prior work with functional interrogation of CFTR-S737A and S768A suggested that phosphorylation of both sites may be inhibitory (Csanady et al., 2005; Wilkinson et al., 1997), but both sites have also been suggested to be stimulatory based on recordings of the same mutants in different experimental contexts (Bozoky et al., 2017; Hegedus et al., 2009). Those results thus cannot be distinguished from ours on the basis of differences in molecular strategy (alanine mutation versus photo-decaging). The total physiological regulation of these sites may be complex, as they have been proposed to interact with other kinases and accessory proteins in addition to their well-demonstrated phosphorylation by PKA (Bozoky et al., 2017; King et al., 2009). Thus, our data speak only to the functional result of PKA phosphorylation of these sites, and only within mammalian cells, where phosphorylation appears to be stimulatory for S737 but inhibitory for S768. The observed kinetics of current change are also in good agreement with apparent rates of S700, S737, and S768 phosphorylation in vivo*,* as measured via phospho-specific antibodies for these sites, in as much as when using the biochemical method, phosphorylation was essentially complete for those sites by the first time point taken after addition of forskolin (∼2 min; Hegedus et al., 2009). In this regard, the decaging strategy presented here is complementary to phospho-biochemical methods since the functional effect of phosphorylation can be observed with high temporal resolution (time scale of seconds).

### Phosphorylation of S795 is critical for CFTR function, in part because it promotes the phosphorylation of S813

**Figure 5.**
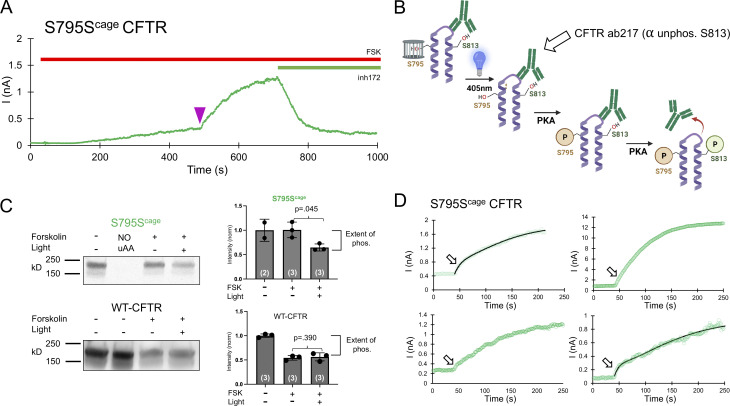
**Decaging and phosphorylation of S795 stimulates channel activity via promoting phosphorylation of S813. (A)** Representative decaging profile for S795S^cage^ CFTR. Delivery of the 250 ms pulse of LED is indicated as a purple arrowhead. **(B)** Schematic explanation of whole-dish photochemistry experiment. **(C)** Left: Western blot using an antibody (CFF ab217) that detects the level of non-phosphorylated S813 motif in the population of CFTR molecules. The lane labeled NO uAA contains lysate from a parallel control transfection without Ser^cage^ AA present in media and shows the expected lack of full-length channel expression. Right: Western blot quantification and significance comparing three independent experiments for stimulated conditions. P values are from unpaired Student’s *t* test, and *n* is the value of experimental replicates indicated in parentheses. **(D)** Depiction of decaging interval (250 s total, with 40 s before the light pulse) of the 4 S795S^cage^ experiments. Arrow indicates the location of initial, fast rate of current increase, which varies from prominent (top left, bottom right with fits shown) to subtle (top right, bottom left). Source data are available for this figure: SourceData F5.

Finally, we encoded caged serine at S795, which has been suggested to be stimulatory on the basis of the characterization of the alanine mutation (Winter and Welsh, 1997). Consistent with this, decaging of S795 resulted in a large increase in current comparable with S813 (8.4 ± 4.7-fold, *n* = 4). This increase appeared slow (Fig. 5 A). Although there exists no data on the in vivo phosphorylation rate of S795, its phosphorylation rate in vitro (the free recombinant R domain in solution) is actually quite fast—i.e., comparable to S700 and S737 (Bozoky et al., 2017). This contrasts with the S813 site, whose phosphorylation is relatively slow in vivo (shown in this study and Hegedus et al., 2009) as well as in vitro (Bozoky et al., 2017). A slow rate of phosphorylation of S813 may be rationalized at least in part because its consensus site is not optimized for PKA interaction, for example, it lacks a conserved hydrophobic residue normally following the phosphoserine (Fig. 1 A, bottom). However, S795 lies in an optimized site, making the slow rate of channel increase from decaging surprising. We hypothesized that one possible mechanism by which S795 decaging would cause a relatively slow and large increase in channel activity is that its phosphorylation may promote the phosphorylation of another site—S813 in particular. Given their order in primary sequence, these sites may reside in the same general structural region; however, this area of the protein sequence is poorly resolved in cryo-EM structures of CFTR (Liu et al., 2017). It is conceivable that phosphorylation of S795 might help liberate the C-terminal region of the R domain from the CFTR channel vestibule.

To test this hypothesis, we used a biochemical approach. We encoded caged serine at the S795 site in CFTR expressed in HEK cells and measured the extent of phosphorylation of S813 with a phospho-specific antibody (Hegedus et al., 2009) with and without decaging of S795. We decaged the S795 residue within CFTR in 35-mm dishes of cells via a custom-mounted LED (Fig. S1). The primary antibody used recognizes the non-phosphorylated S813 motif; thus, higher relative phosphorylation is indicated by reduced CFTR signal on the Western blot (Fig. 5 B). Cells were first stimulated by 10 μM forskolin, after which half of the dishes were exposed to blue light, with the other half serving as controls. Finally, all dishes were directly lysed in a cold sample buffer on ice and flash frozen.

**Figure 6.**
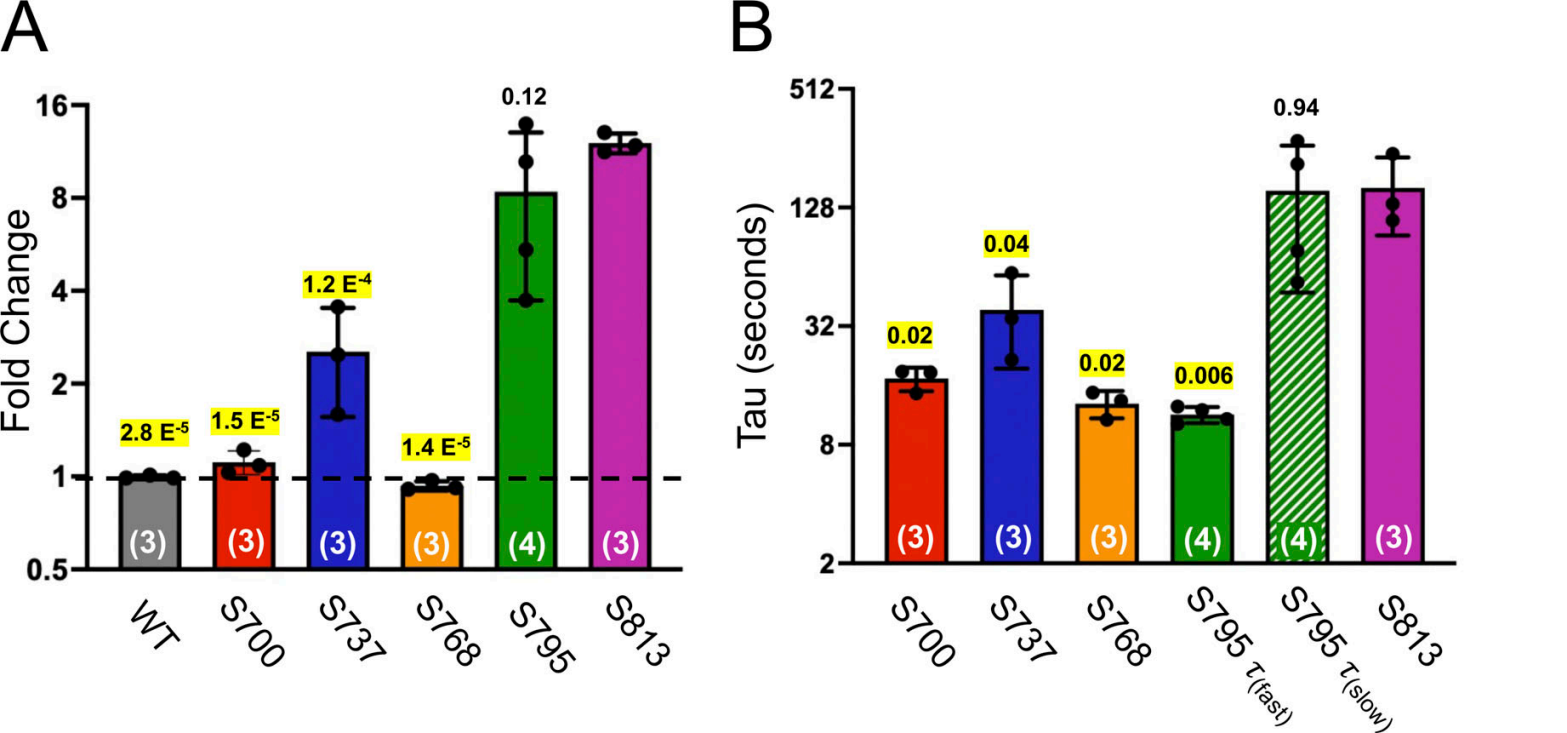
**Summary data of serine decaging profiles for CFTR R domain variants. (A)** Fold change upon LED light pulse applied at the plateau of forskolin activation. Dashed line at the level of no change (i.e., fold change of 1). P values for unpaired *t* tests against S813 data are shown above each column; all except S795 were P < 0.05 and are highlighted as significant. *n* values of experimental replicates are shown in parentheses. **(B)** Rates of increase or decrease in CFTR current from exponential fits (τ) of the decaging phenotypes for all variants wherein serine was decaged. S795 was fit to a double exponential, from which arose fast (solid green) and slow (dashed green) components. P values for unpaired *t* tests against S813 data shown above each column; all except the slow component of S795 increase were P < 0.05 and are highlighted as significant. *n* values of experimental replicates are shown in parentheses.

The Western blots revealed that, in the caged S795S^cage^ condition, even after 10 min of forskolin incubation, S813 was minimally phosphorylated, i.e., the signal was similar to that of the initial, forskolin-free level (Fig. 5 C). By comparison, in dishes wherein S795 was decaged by blue light, S813 was phosphorylated, as indicated by a decreased signal from the antibody. As a control, this experimental protocol was repeated with WT CFTR, and phosphorylation of S813 was shown to be independent of light treatment (Fig. 5 C). The functional decaging phenotype from S813 previously established that the slow phosphorylation of S813 essentially governs the gating of CFTR (Fig. 2). Taken together, the biochemical and functional results from decaging S795 explain both the slow kinetics and the large fold increase in channel current that was observed. Note that there exists an alternative possibility that the presence of the caged serine side chain at position 795 may somehow impede the phosphorylation of S813. While this cannot be formally excluded, it is highly unlikely since both sites are phosphorylated (and thus tolerate the profound structural addition of PO_4_^2−^) in vivo. Finally, we observed upon close inspection of the S795 traces that the decaging phenotype for S795 appeared biphasic (Fig. 5 D). The rate of the first variable (τ of 11.4 ± 1.1 s, *n* = 4) closely matched the average rate of the fast PKA consensus sites S700 and S768 (Fig. 4), while the rate of the second, larger phase of current increase (155.6 ± 108.2 s, *n* = 4) resembled that of decaging and phosphorylation of S813 (161.0 ± 68.8 s; Fig. 2 and Fig. 6).

## Discussion

In this study, we utilized a chemical biology approach to characterize the functional effects of making single sites in the CFTR R domain available for phosphorylation in vivo. Our characterization of binding kinetics via SPR suggests that the cage not only prevents phosphorylation but also prevents PKA binding. Diverse effects were observed, from small, fast effects to large, slow stimulation of channel activity.

Despite their small effect, the “fast” sites may be generally informative to an understanding of the intrinsic kinetics of cellular PKA signaling since, by using channel activity as output, the flash decaging experiment is designed to reveal the nearly immediate effect of making a single site available to PKA. The S768 motif is optimized for PKA interaction, and its ultrafast rate (τ ∼13 s) may represent a sort of “maximal kinetic response” within this system. We can also speculate further, however, that such a constant may approximate an upper limit for the speed of PKA signaling in the presence of strong in vivo stimulation of a human cell line.

The effects of phosphorylation by S813 or the promotion of its phosphorylation by S795 suggest that it serves as an important regulator of channel function, while some other sites such as S700, S737, and S768 may play supportive roles in the fine-tuning of activity. This begs the question: why is the S813 site not optimized for PKA interaction? It specifically lacks the conserved hydrophobic residues that usually follow the phosphorylated serine in so-called “strong” PKA consensus sites and instead bears a glutamine and glutamate, both unfavored in the motif (Phosphosite Plus v. 6.6.0.2). An alignment of orthologous R domain sequences around S813 reveal that it is highly conserved among jawed vertebrates but absent in sea lamprey, suggesting it may have emerged relatively late in evolution (Infield et al., 2021). However, this late emergence does not explain its lack of optimization, for example, S700 was also absent in lamprey but is fully optimized in mammals (Infield et al., 2021).

Although S813 is slow to be phosphorylated, the relative extent of its phosphorylation in vivo is reported to be strong (comparable with or greater than that of other sites; Cheng et al., 1991; Hegedus et al., 2009), in response to PKA stimulation. The data from our study and others thus lead to the conclusion that the S813 motif is coded into CFTR regulation to respond fully*,* but only to strong intracellular signaling, as governed by its unoptimized consensus motif and by phosphorylation at S795. S795 may act as a “ready” signal—its phosphorylation indicates a durable rise in cAMP and prolonged activation, which allows S813 to be fully phosphorylated to activate the CFTR channel. It is unlikely that CFTR is the only system that utilizes this type of mechanistic control: other proteins may be similarly tuned to respond with proper temporal control in their respective cellular contexts. This leads us to wonder how divergence from consensus within the PKA motifs of other substrates may contribute to their functions. For example, despite an obvious “need for speed” intrinsic to the adrenergic flight-or-fight system in cardiomyocytes, only one of the four putative phosphoserines in the relevant phosphoprotein (the regulatory GTPase Rad) resides in an optimized PKA consensus site (Liu et al., 2020).

In this report, we describe a chemical biology approach that enabled the real-time functional interrogation of single phosphorylation sites in CFTR expressed in mammalian cells. Future studies may combine this method with conventional mutations to alter other amino acids in these PKA consensus sites and observe how decaging phenotypes (and thus, the function of individual sites) are affected. We also note that the rates observed likely reflect not only the action of PKA (which is dominant under stimulation by forskolin) but any counteracting action from endogenous phosphatases. The precise effect of these may be elucidated in future studies using targeted phosphatase inhibitors. More broadly, this system may potentially be extended to glean position-specific insights into the phosphoregulation of myriad other membrane proteins whose phosphorylation is intimately coupled to function.

## Supplementary Material

SourceData F2 is the source file for Fig. 2.

SourceData F4 is the source file for Fig. 4.

SourceData F5 is the source file for Fig. 5.
