# Supplementary material for: Real-time observation of functional specialization among phosphorylation sites in CFTR
Source: J Gen Physiol. 2023 Jan 25;155(4):e202213216. doi: 10.1085/jgp.202213216 (PMC9930130; doi:10.1085/jgp.202213216)
Supplement: SourceData F2 — is the source file for Fig. 2. [file JGP_202213216_SourceDataF2.pdf]

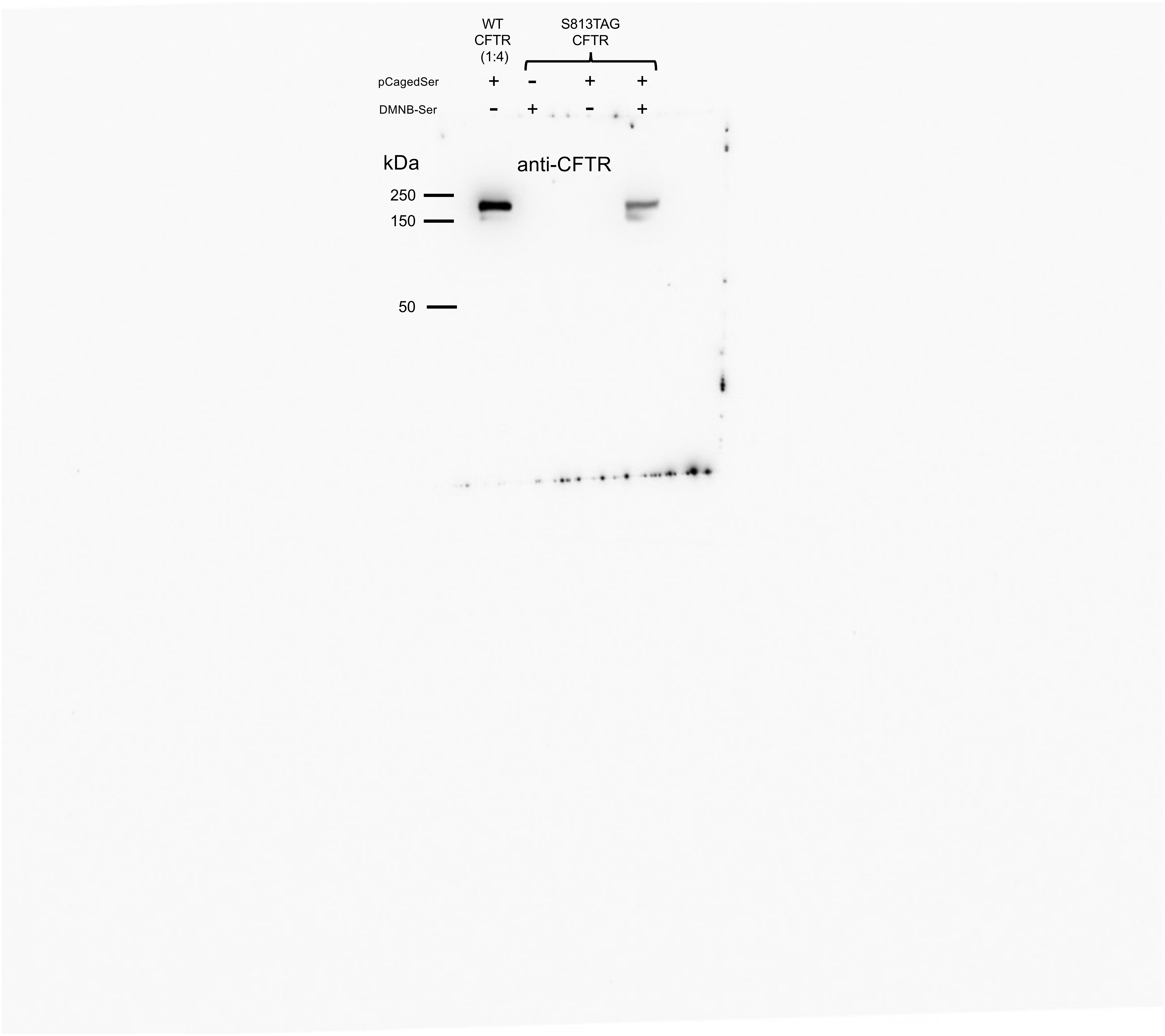

Additional File Fig 2_1: Uncropped membrane image for the CFTR signal.

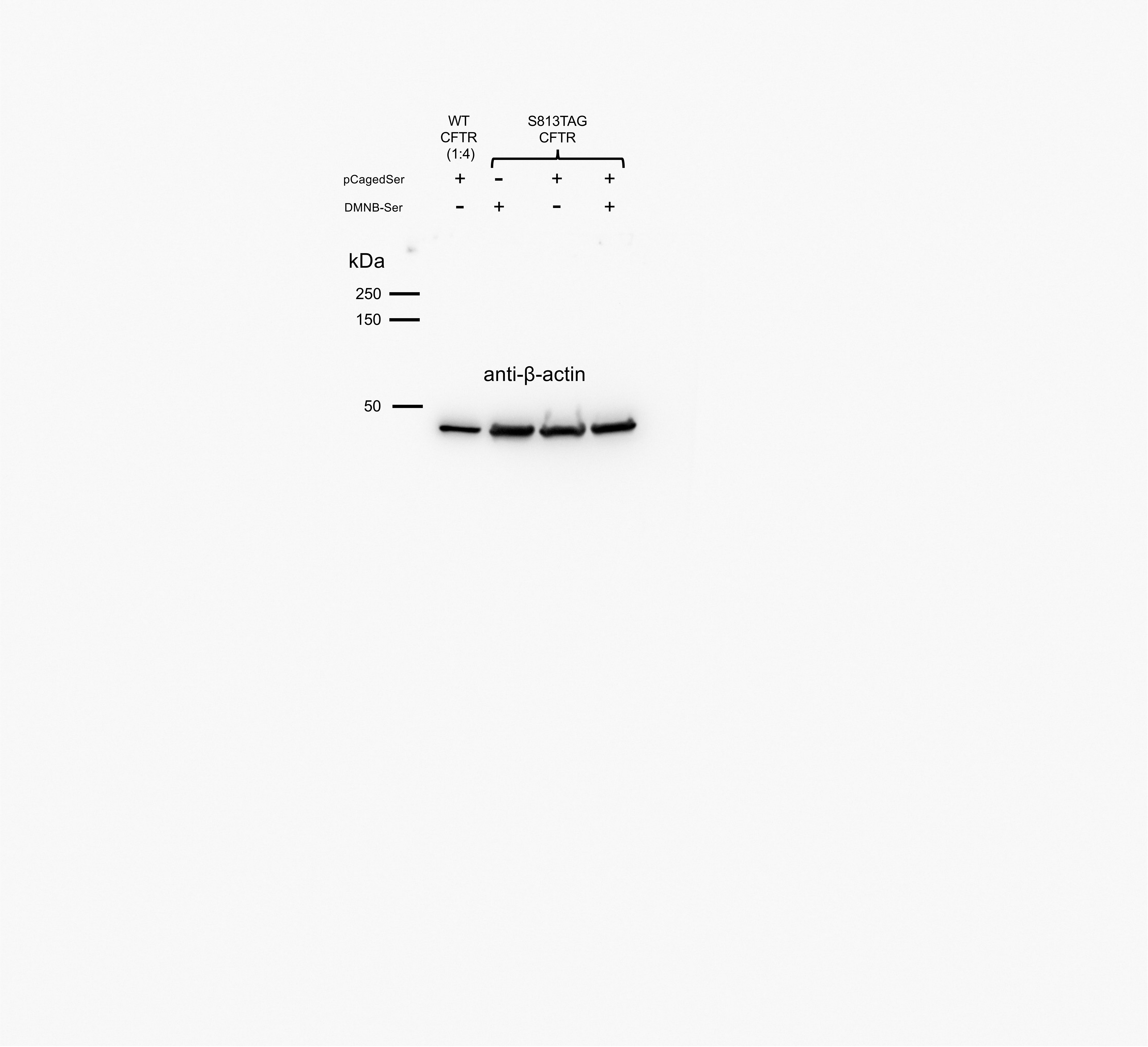

Additional File Fig 2_2: Uncropped membrane image for the beta-actin signal.
