# Supplementary material for: Real-time observation of functional specialization among phosphorylation sites in CFTR
Source: J Gen Physiol. 2023 Jan 25;155(4):e202213216. doi: 10.1085/jgp.202213216 (PMC9930130; doi:10.1085/jgp.202213216)
Supplement: SourceData F5 — is the source file for Fig. 5. [file JGP_202213216_SourceDataF5.pdf]

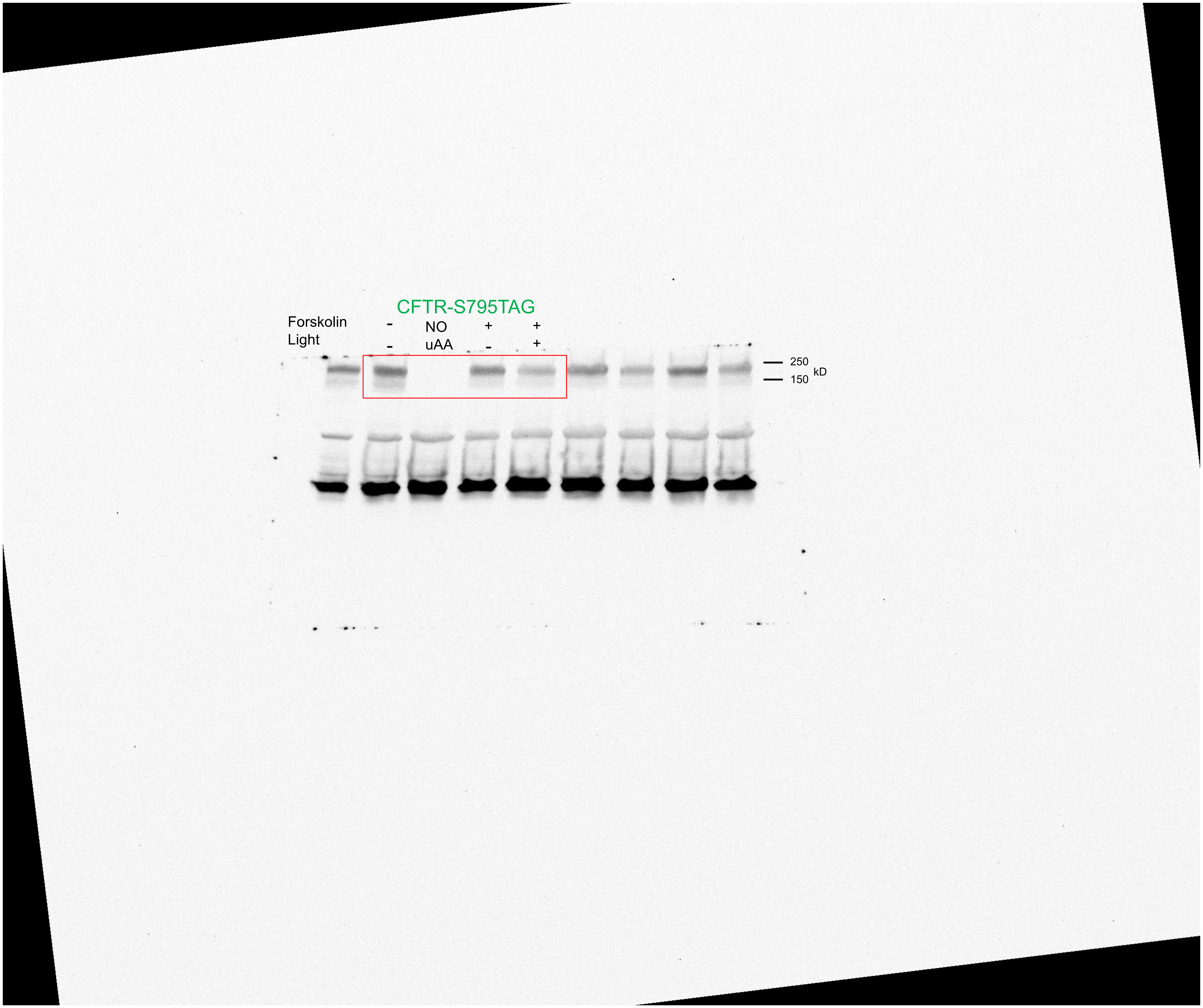

Additional file Fig 5_1: uncropped blot associated with Figure 5C (top). Region of samples shown in main figure is bounded in red. Note that the background bands are present in WT expression as well as in the mutant (compare to additional file Fig 5_2). In these experiments, cells were lysed directly in cold LSB, which has the effect of more background bands showing up on western blot compared to RIPA lysis + centrifugation. No uAA denotes no caged-Ser amino acid was added.

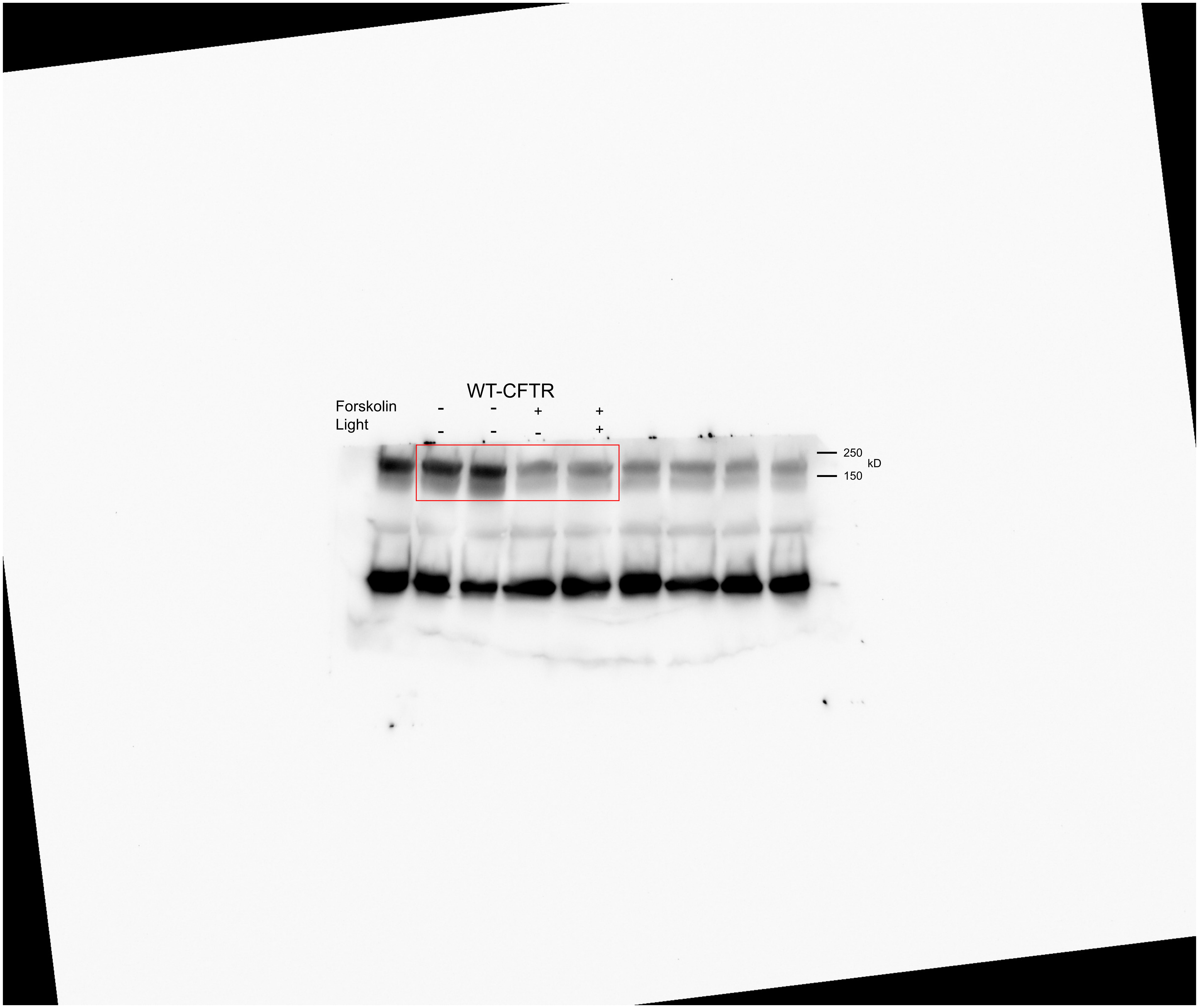

Additional file Fig 5_2: uncropped blot associated with Figure 5 C (bottom). Region of samples shown in main figure is bounded in red. Note that the background bands are present in WT expression as well as in the mutant (compare to additional file Fig 5_1). In these experiments, cells were lysed directly in cold LSB, which has the effect of more background bands showing up on western blot compared to RIPA lysis + centrifugation.
